# 3D Engineered Biomimetic Platform for Characterization of Collective Invasion, Tumor Emboli Formation, and Lymphatic Dissemination

**DOI:** 10.1109/OJEMB.2026.3666977

**Published:** 2026-02-23

**Authors:** Caroline Way, Ralph F. Erdmann, Larisa Gearhart-Serna, Pritha Pai, Natalia Roman, Bruce Klitzman, Gayathri R. Devi

**Affiliations:** Division of Surgical SciencesDepartment of SurgeryDuke University School of Medicine 12277 Durham NC 27710 USA; Department of Biomedical EngineeringDuke Pratt School of Engineering Durham NC 27708 USA; Duke Consortium for Inflammatory Breast CancerDuke Cancer Institute 14409 Durham NC 27710 USA; Division of Surgical SciencesDepartment of SurgeryDuke University School of Medicine 12277 Durham NC 27710 USA; Duke Consortium for Inflammatory Breast CancerDuke Cancer Institute 14409 Durham NC 27710 USA; Division of Surgical SciencesDepartment of SurgeryDuke University School of Medicine 12277 Durham NC 27710 USA; Duke Consortium for Inflammatory Breast CancerDuke Cancer Institute 14409 Durham NC 27710 USA; Department of SurgeryMedical Unversity of South Carolina Charleston SC 29425 USA; Department of Biomedical EngineeringDuke Pratt School of Engineering Durham NC 27708 USA; Duke Consortium for Inflammatory Breast CancerDuke Cancer Institute 14409 Durham NC 27710 USA; Department of Biomedical EngineeringDuke Pratt School of Engineering Durham NC 27708 USA; Division of Plastics and Reconstructive SurgeryDuke University School of Medicine 12277 Durham NC 27710 USA; Duke Consortium for Inflammatory Breast CancerDuke Cancer Institute 14409 Durham NC 27710 USA; Division of Surgical SciencesDepartment of SurgeryDuke University School of Medicine 12277 Durham NC 27710 USA; Department of Surgery, Hollings Cancer CenterMedical University of South Carolina 2345 Charleston SC 29425 USA; Duke Consortium for Inflammatory Breast CancerDuke Cancer Institute 14409 Durham NC 27710 USA

**Keywords:** Lymphovascular invasion (LVI), tumor emboli, collective migration, metastasis, inflammatory breast cancer (IBC), 3D-printing, additive manufacturing

## Abstract

*Goal:* Emerging evidence in diverse tumor types establishes a link between lymphatic dissemination and collective tumor cell invasion. To simulate the biomechanical features of the tumor-lymphatic microenvironment, we developed a 3D tumor-lymphatic architecture biomimetic (T-LAB) platform. *Methods:* Mathematical and computational fluid dynamics modeling were used to determine the fluid flow, oscillatory flow-induced shear stress, and system pressure in the 3D-printed macrofluidics platform. *Results:* Various human breast cancer cell lines and human dermal lymphatic endothelial cells (HDLEC) were seeded on a matrix in the T-LAB and imaged for up to 96 h to assess cell morphology, viability, migration, and invasion. Co-culture of inflammatory breast cancer cells with HDLEC in the T-LAB, determined to simulate the fluidic properties of the tumor lymphatic microenvironment, demonstrated tumor cell clusters/emboli formation and collective invasion similar to the clinicopathological features observed in patients. *Conclusions:* The 3D T-LAB model developed here can be used to culture any type of tumor cell to study topographical features that impact tumor-lymphatic interface, collective invasion, and lymphatic dissemination.

## I. Introduction

**Fig. 1.**
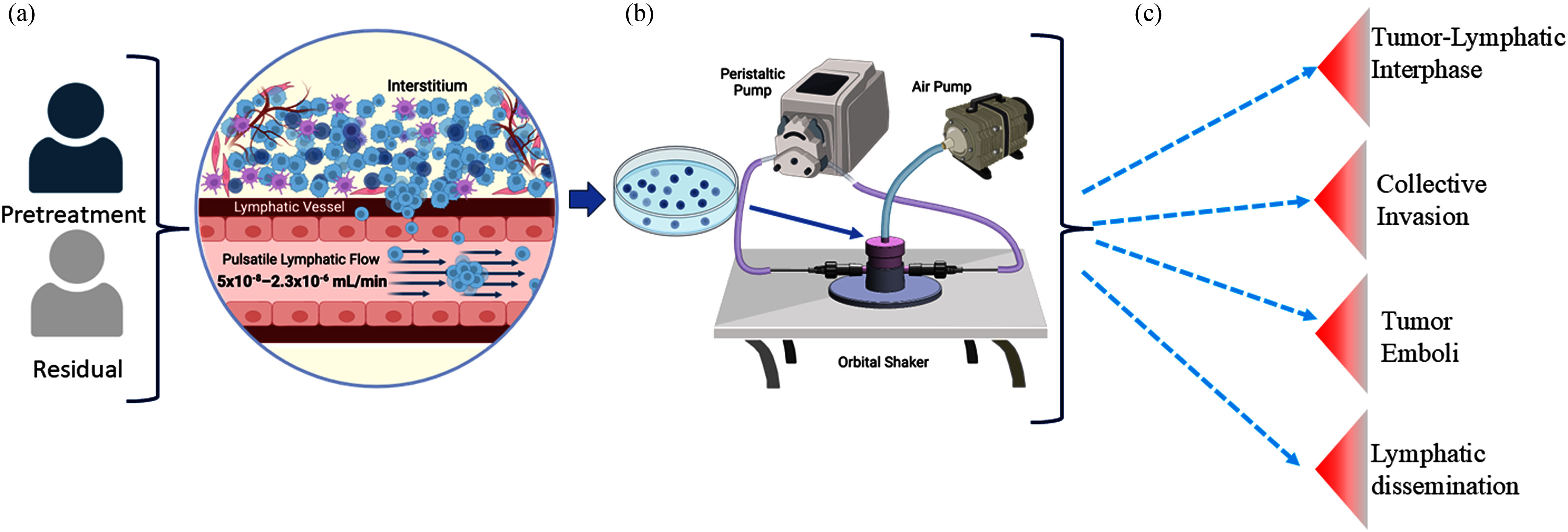
Schematic representation of the 3D T-LAB platform and workflow. (a) Patient derived samples including pretreatment or post-treatment residual tumor cells and microenvironment components can be used as source material to isolate and culture. (b) Components include – the 3D printed module, orbital shaker, peristaltic pump, and air pump. (c) Potential biological applications of the biomimetic platform.

Metastasis occurs when a primary tumor gains the ability to move by breaking down surrounding proteins in the matrix and stroma. It then enters the bloodstream or lymphatic vessels [1], [2], [3], [4]. While lymph nodes are the most common sites in cancer metastasis, it was previously believed that lymphatic spread was a passive process. However, recent evidence suggests that tumor cells interact dynamically with lymphatic endothelial cells, triggering signaling mechanisms that promote both entry into and movement within lymphatic vessels [4], [5], [6]. Moreover, tumor cells that survive within lymphatic vessels often stop at secondary sites, forming hidden or inactive micro-metastases. These can later reappear, causing tumor relapse and worsening survival rates [5], [6], [7], [8], [9]. Because lymphovascular invasion is known to be an independent predictor of survival in several types of cancer [5], [7], [10], [11], [12], [13], [14], such as breast, thyroid, colon, lung, and melanoma, it has become a target for drug development. However, there is an unmet need for models that accurately mimic this complex process [15]. Although two-dimensional (2D) cell culture models are highly controllable, they do not recapitulate the 3D organization and function of lymphatics in vivo. Furthermore, developing animal models including our recently described transgenic mice to study lymphatic metastasis face challenges in the ability to distinguish the relative contributions of the biological and biophysical factors of this complex pathophysiological microenvironment [16], [17], [18], [19]. These limitations are a critical roadblock for the adoption of personalized medicine in oncology since the inhibition of tumor cell motility and cell migration is a common feature of many drugs currently in cancer clinical trials. Therefore, there is a clear, unmet need for preclinical models to study the tumor interstitium, tumor-lymphatic vessel interface, and metastatic dissemination in a single platform which has been identified in previous studies [20], [21]. In this study, we engineered a 3D biocompatible and autoclavable template that supports culturing cells using conditions that simulate the biochemical and physiological characteristics of the tumor lymphatic microenvironment (Fig. 1).

## II. Materials and Methods

### A. Cell Culture and Histology

MDA-MB-231 (triple negative), SUM149 (IBC), and MDA-IBC3 (PDX) [22] were labeled with green fluorescent protein (GFP) or red fluorescent protein (RFP) as previously described [19] and cultured according to established protocols [22], [19]. Adult human dermal lymphatic endothelial cells (HDLEC) (Promocell, Germany) were cultured according to manufacturer instructions (details in the supplementary methods). A deidentified tissue histology section from an IBC patient was used in this study with prior patient consent and approval from the Duke University Institutional Review Board.

### B. Dermal Lymphatic Capillary Flow Rate

Lymphatic capillary flow rates were calculated (Fig. 1(a)) from the reported average dermal lymphatic velocity of 10 µm/s [23] and lymphatic capillary diameter range of 10 – 70 µm [16], [17], [24] using (1):

\begin{equation*} Q = Av \tag{1} \end{equation*}

where $Q$ is flow rate, $A$ is the cross-sectional area, and $v$ is the average velocity of the fluid. The vessel was assumed to be cylindrical, and the cross-sectional area was calculated using radius $r$ as

\begin{equation*} A = \ \pi {{r}^2} \tag{2}
       \end{equation*}

### C. Fabrication of the 3D T-LAB

**Fig. 2.**
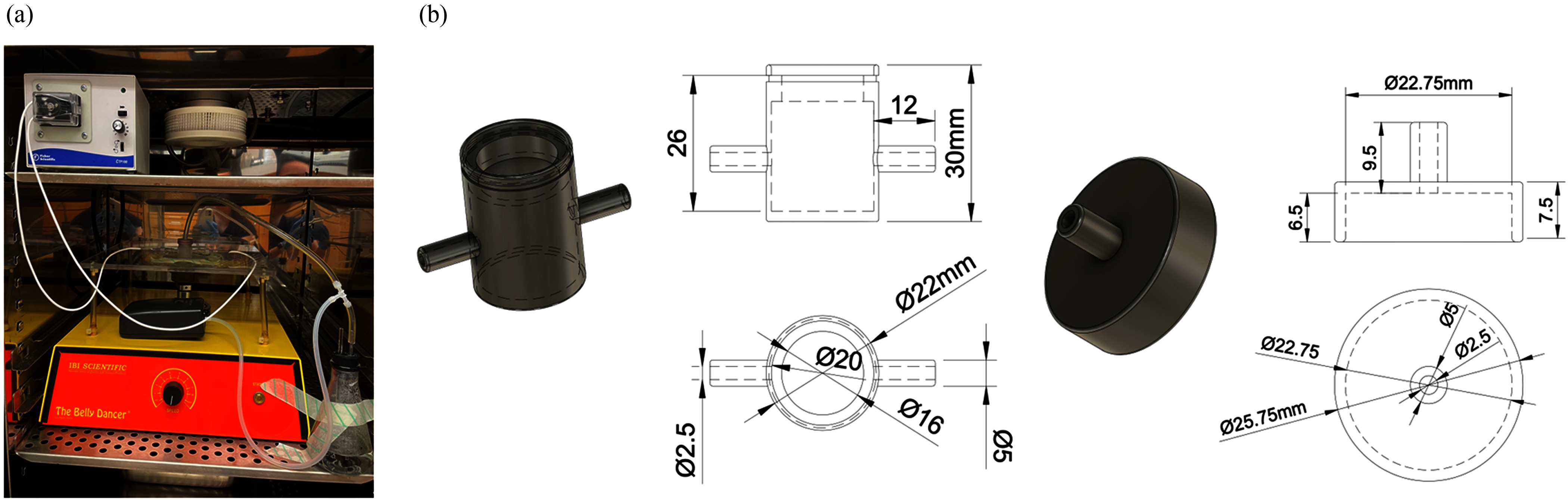
3D T-LAB platform component. (a) 1x photographic image (taken on handheld 12 MP, ƒ/2.2 front lens camera) of the 3D T-LAB platform – the biomimetic platform, orbital shaker, peristaltic pump, and air pump with pressure regulator in a cell culture incubator at 37deg C. The biomimetic system was placed in a custom printed mount adhered to the platform of an orbital shaker that is set to a rotation speed of 40 rpm. (b) Dimensional drawings of the 3D biomimetic chamber template, consisting of a bottom chamber (*Top*) and lid (*Bottom*).

The 3D T-LAB Platform consists of a 3D biomimetic platform, orbital shaker, peristaltic pump, and air pump with a pressure regulator (Figs. 1(b), 2(a)). To simulate lymphatic vasculature, which are tubular structures in a tumor microenvironment, the primary module was designed in CAD software (Autodesk Fusion 360 2.0.16490, San Francisco, CA, US; SolidWorks 2022 SP3, Dassault Systèmes, Velizy-Villacoublay, Ile-De-France, France). This includes a top chamber consisting of a transwell representing the tumor stroma set within a bottom chamber representing a lymphatic vessel with fluid flow capacity. These templates were 3D printed with biocompatible and autoclavable resin (Biomed Clear, Formlabs, Somerville, MA, US). Each template **(** Fig. 2(b)) consists of a main cylindrical chamber (inner diameter: 20mm, height: 30 mm) that fits a removable transwell insert (6.5 mm with 8.0 µm pore, Corning, Corning, NY, US) suspended 12 mm above the bottom of the chamber, which can be removed for imaging at various timepoints. Templates were oriented for printing in a way that did not require internal supports in order to create a smooth interior discouraging cell build up or irregularities between templates. The printed templates were washed in isopropyl alcohol for 15 minutes, air-dried for at least 30 minutes, and then cured for 60 minutes at 60 °C.

### D. Pressure Configuration

An air pump with a custom pressure regulator was attached to the template lid, which maintains a constant elevated pressure (Fig. 1(b)). An O-ring coated in high vacuum grease were used to create an air-tight seal between the lid and main body of the template such that 1) pressure can be manipulated to and maintained at non-atmospheric values and 2) media and cells cannot leak out. Airtightness was evaluated through template submersion tests for the presence of a steady stream of bubbles in the pressure regulator.

To calculate the increase in pressure in the prototype for the given volume of water in the custom pressure regulator, (3) was used [25],

\begin{equation*} {{P}_g} = \rho gh \tag{3} \end{equation*}

wherein ${{P}_g}$ is gauge pressure, $\rho $ is density of water (993.36 kg/m^3^), $g$ is the gravitational constant (9.81 m/s^2^), and $h$ is the distance between the submerged pipette in the pressure regulator and the water’s surface [26].

### E. Fluid Flow and Oscillatory Force Configuration

An external engineering-grade peristaltic pump (FH10, Fisherbrand, Waltham, MA, US) was attached with 0.085” (2.2 mm) O.D., 0.04” (1 mm) I.D. silicon tubing (Silastic, Midland, MI, US) to the main chamber of the templates. A spout on each side of the biomimetic platform (5 mm O.D, 2.5 mm I.D.), centered 10 mm above the bottom of the chamber, fits a male luer lock to 1/4;” hose barb adapter (Masterflex, Radnor, PA, US) attached to a Luer-Lok™ Syringe Needle, 16 g x 1’ (Thomas Scientific, Chadds Ford, PA, US). Both needles were then connected to 0.085” O.D., 0.04” I.D. silicon tubing (Silastic, Midland, MI, US) creating a complete path for circulation. The tubing feeds into the biomedical/engineering grade peristaltic pump (FH10, Fisherbrand, Waltham, MA, US) with the pump set to an average flow rate of 0.642 mL/min (STD = 0.003 mL/min). The biomimetic system was placed in a custom printed mount adhered to the platform of an orbital shaker that is set to a rotation speed of 40 rpm (Fig. 2(a)).

### F. Characterization of the 3D Biomimetic Platform Viscosity and Density

To measure kinematic viscosity of each culture media used in the biomimetic, we customized a system wherein a Cannon Semi Micro Viscometer (CMSMC-25, Cannon Instrument Company, Cannon Instrument Co., State College, PA) with a calibrated range of 0.4 to 2 cSt was submerged in a 3.5 L beaker of water placed inside a water bath maintained slightly over 37 °C (to accommodate for any heat loss) using a circulating water pump (1122s, VWR, Randor, PA, US). Water was used as the standard for kinematic viscosity calibration (0.002302 mm^2^/s^2^ at 37 °C) and multiplied by recorded time for each culture media tested to determine the kinematic viscosity. In order to find the absolute (dynamic) viscosity of the media, (4) was used [27],

\begin{equation*} v = \mu /\rho \tag{4} \end{equation*}

where $v$ is kinematic viscosity of the media (cSt), $\mu $ is absolute (dynamic) viscosity (cP), and $\rho
      $ is density (g/mL). The solved absolute viscosity was then applied to later shear stress calculations (5). To determine media densities (g/mL), the media were warmed to 37 °C using the same method. 1 mL of each was then weighed using an analytical balance (Denver Instruments, Bohemia, NY, US), and the mass was divided by volume.

### G. Computational Fluid Dynamics Modeling

Computational fluid dynamics modeling (CFD) was conducted using the SolidWorks Flow Simulation package included in the SolidWorks software (Dassault Systèmes, Velizy-Villacoublay, Ile-De-France, France). The 3D CAD model imported for the simulation (in which all fluid-air boundaries were represented as a solid surface) was prepared in Autodesk Fusion 360 (Autodesk Fusion 360 2.0.16490, San Francisco, CA, US). A 3D flow simulation was then run using the established flow rate as an input parameter (Fig. 4).

### H. Shear Stress

Within the biomimetic system, two sources contribute to the total shear stress experienced by tumor cells: 1) fluid flow, driven by the peristaltic pump, and 2) additional shear stress applied by the orbital shaker. In order to analytically approximate the shear stress contributed by the orbital shaker, a value for the maximum wall shear stress was calculated with (5) [28]:

\begin{equation*} {{\tau }_{max}} = \ \alpha \sqrt {\rho \mu {{{\left(
       {2\pi f} \right)}}^3}} \tag{5} \end{equation*}

where $\alpha $ is the radius of rotation for the orbital shaker (2.54 cm), $\rho $is the density of the fluid (0.9899 g/mL), $\mu $is the dynamic viscosity of the fluid (0.7124 cP), and $f$is the frequency of rotation of the orbital shaker (40 rpm).Though the orbital shaker does not apply uniform shear stress, at lower frequencies (like the 40 rpm used for the biomimetic system) the shear stress is relatively stable and can be treated as near-uniform [28]. Additionally, while (5) is a well-cited analytical approximation for orbital-shaker setups, Ferrell et al. [28] suggest that at lower frequencies, the equation overestimates actual shear stress by two-fold. Therefore, the resultant value was also halved and compared to target shear stress ranges.

### I. Preparation of the 3D T-LAB Co-Culture System

A transwell with 8.0 μm pores was placed into the shelf of the 3D T-LAB bottom chamber and hydrated with serum-free media for 1 hour, after which the media was removed. For SUM149 quantitative viability and HDLEC-omitted experiments, no HDLEC layer was added. For all other experiments, after membrane rehydration and removal, 3.5x 10^5^ HDLEC cells were seeded atop the transwell permeable membrane. 66.4 µL of collagen was then poured atop the membrane or HDLEC layer and solidified for 1 hour at RT to make a semi-solid 2 mm layer. On top of the collagen layer, 2.5 × 10^5^ breast cancer cells were seeded in 250 µL of serum-free media or media supplemented with PEG8000 for IBC cell lines. Serum-free media without tumor cells was used for HDLEC quantitative viability studies. The bottom chamber was then connected on both sides via tubing to the peristaltic pump, and media was added to fill the tubing and bottom chamber to the level of the transwell membrane. The peristaltic pump was set to the lowest speed, and media flow was visually confirmed. The 3D T-LAB lid was then fixed and tightly sealed to the bottom chamber and connected to the air pump. The entire system with attached pumps was placed on an orbital shaker at 40 rpm inside an incubator. The detailed method of imaging and viability assay can be found in the supplementary methods.

### J. Characterization of Disseminated Tumor Cell Clusters

In select experiments, the invaded or disseminated tumor cell clusters were collected from the lymphatic chamber media. In comparative experiments, the collected cell clusters were expanded in culture and imaged alongside tumor cells not used in the 3D T-LAB, using the 20X objective in the EVOS M7000 inverted microscope.

## III. Results

### A. Characteristics of the 3D T-LAB

**Fig. 3.**
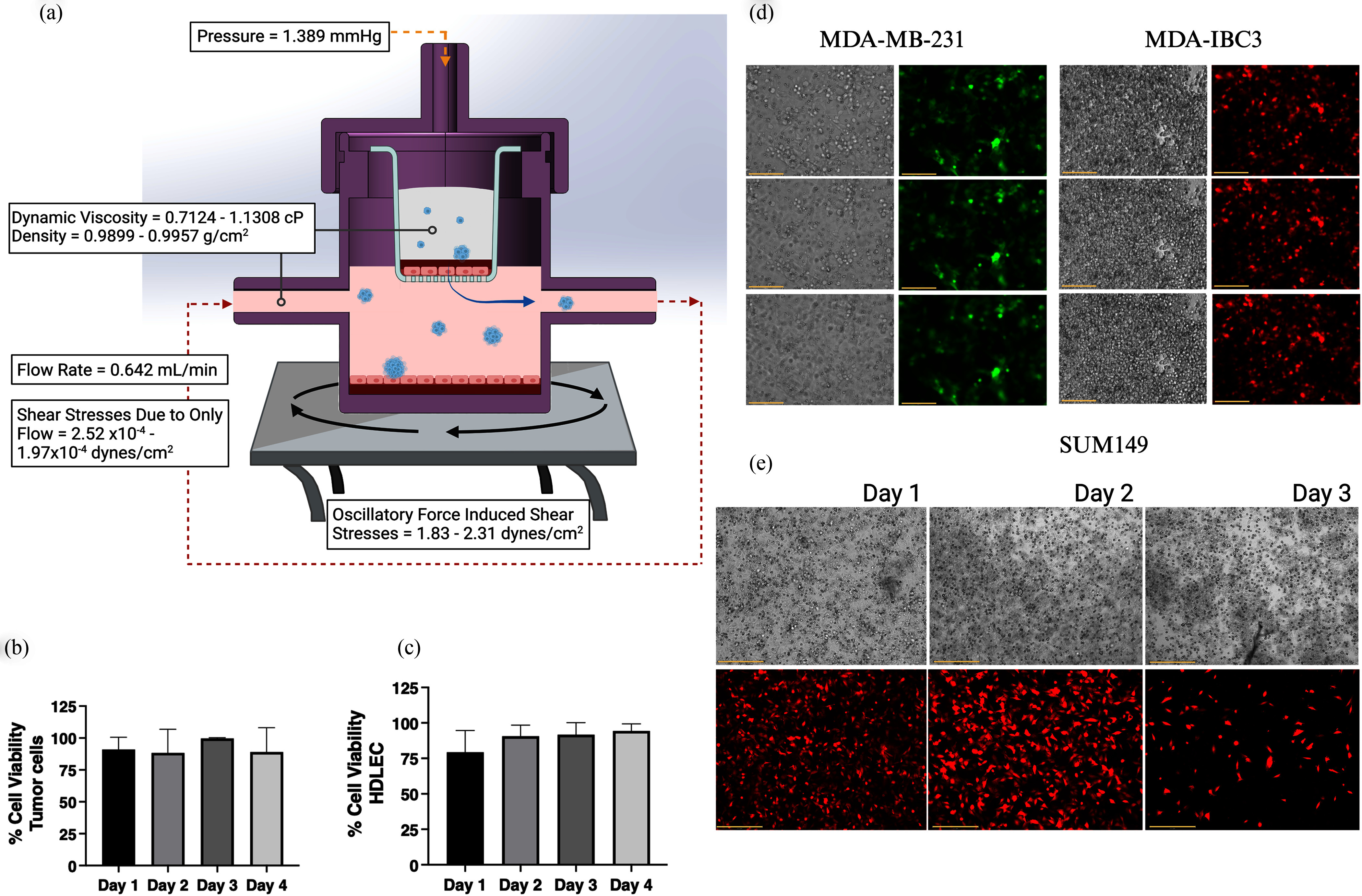
Characterization of 3D T-LAB. (a) Measurement of critical biomechanical parameters of fluid flow, shear stress, and pressure present in the 3D T-LAB to simulate the lymphatic environment. Fluid flow of media in the bottom chamber is driven through tubing by a peristaltic pump and simulates the flow of the dermal lymphatic capillaries. Maximum shear stress experienced at the bottom of the transwell due to only this flow (a stationary environment) was calculated using a computational fluid dynamics model. To mimic lymphatic shear stress, additional shear stress is applied to the 3D T-LAB with an orbital shaker, and the maximum shear stress contributed by these oscillatory forces was analytically approximated. Within the transwell chamber, the permeable membrane represents the basement layer, while a layer of lymphatic endothelial cells seeded on the membrane simulates the lymphatic endothelium. A collagen layer is added atop the lymphatic cell layer to represent the breast interstitium. The final layer, atop the collagen, houses the tumor cells in media. To simulate the increased pressure of the tumor interstitium, an air pump attached at the lid supplies a constant pressure and establishes a pressure gradient downward through the transwell chamber into the lymphatic chamber, similar to the draining pressure characteristic of many lymphatic vessels. Cell viability in the transwell/upper chamber, including cells atop the collagen, in the collagen matrix, and on the permeable membrane, over 4 days shown for (b) SUM149 tumor cells and (c) HDLEC. (d) Images from modulated flow and rotational culture conditions showing GFP- or RFP-tagged tumor cells that have invaded the collagen layer as z-stacks at the transwell-HDLEC interface at a single time point or (e) over 0-3 days. Bar represents 150µm in all images shown.

The 3D T-LAB Platform consists of a 3D module for cell culture (Fig. 2(b)), orbital shaker, peristaltic pump, and air pump with a pressure regulator (Fig. 1(b)). To mimic the higher pressure observed in the tumor interstitium, the biomimetic platform is connected to an air pump that maintains an absolute pressure of 185.152 Pa, which is 1.389 mmHg above atmospheric pressure. Moreover, a transwell containing a collagen hydrogel layer and a monolayer of HDLEC divides the two chambers of the biomimetic platform recapitulating the tumor lymphatic vessel interface. The air pump pressure is exerted through these layers resulting in a draining pressure into the lymphatic fluid flow below, which is physiologically appropriate. Based on previously published studies characterizing the anatomy of lymphatic vessels [16], [17], [24], we calculated that the physiological propulsion flow rate in the dermal lymphatic vessels would be in the range of 5x10^-8^ mL/min – 2.3 × 10^-6^ mL/min **(** Fig. 1(a)). Taking in to account the size constraints of our biomimetic platform, which is on a much larger scale and non-cylindrical, we first determined the hydraulic diameter at various sections to be in the average range of 17.11 mm and then used the lowest speed settings of a peristaltic pump, which can withstand the humid environment of the cell culture incubator at 37 °C, to achieve a flow rate of 0.642 mL/min in the biomimetic platform. Finally, media used for cell culture alone or with PEG8000 were determined to exhibit kinematic viscosity (0.7196 – 1.1356 cSt), dynamic viscosity (0.7124 – 1.1308 cP) and density (0.9899 – 0.9957 g/mL) that closely simulate the characteristics of biological lymphatic fluid (Fig. 3(a); **Supplementary Table 1**) [29], [30].

### B. Assessing Cell Invasion

In proof of principle studies to evaluate the biomimetic platform’s function, various breast cancer cell lines (SUM149, MDA-MB-231), patient derived explant cells (MDA-IBC-3), were seeded in the top chamber of the biomimetic platform and images were acquired by live imaging using EVOS microscopy. Cell viability datasets (Fig. 3(b), (c)) from different time points reveal that both tumor cells (Fig. 3(b)) and HDLEC (Fig. 3(c)) exhibit an average of 75-80% viability when cultured in the transwell inserted in the 3D T-LAB. Furthermore, z-stack images of breast cancer cells at 24 h show viable cells in the basement layer simulating the tumor-lymphatic interface (Fig. 3(d)) along with the ability to image the cell invasion and migration patterns over time (Fig. 3(e)).

### C. Modeling Velocity and Shear Stress Profiles

**Fig. 4.**
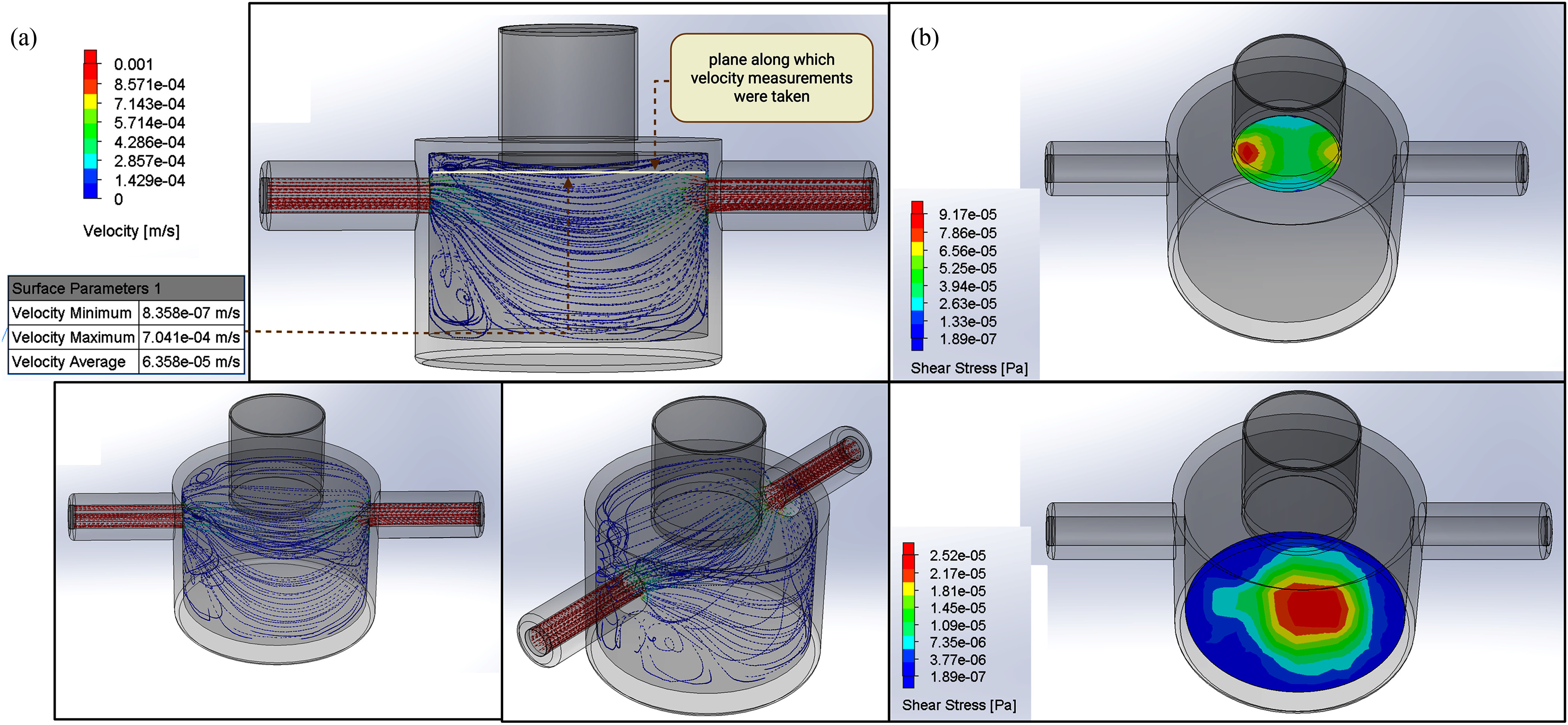
Fluid flow simulation of the 3D T-LAB model. Assumptions for the flow simulation included a steady-state, incompressible flow governed by the Navier-Stokes equations for conservation of momentum and the continuity equation for conservation of mass. Flow at the inlet was assumed to be fully developed and defined with a flow rate of 0.642 mL/min. The media used in the biomimetic platform was modeled as media at 37 °C (dynamic viscosity: 0.7124 cP, density: 0.9899 g/mL). The turbulence parameter was 2%, and the boundary condition for the outlet was set as open to the environment. The no-slip boundary condition was applied to all walls. (a). The velocity profile of media flow through the biomimetic platform, shown here in three perspective views, was generated using the SolidWorks Flow fluid flow simulation model. Velocity measurements taken along the plane 0.5 mm below the bottom of the transwell indicate that the velocity varies between 8.358x10^-7^ and 7.041x10^-4^ m/s with an average of 6.358x10^-5^ m/s, while a maximum velocity of 0.004 m/s is reached in both spouts on either side of the biomimetic platform. (b). A heat map of shear stresses experienced at the transwell surface (*Top*) and at the bottom of the biomimetic platform (*Bottom*) due to only the media flow, was also generated using the SolidWorks Flow fluid flow simulation model. The maximum shear stress at the transwell surface is 9.17x10^-5^ Pa or 9.17x 10^-4^ dynes/cm^2^, while the maximum shear stress at the bottom of the biomimetic platform is 2.52x10^-5^ Pa or 2.52 x10^-4^ dynes/cm^2^.

Next, we used computational fluid dynamics (CFD) modeling to estimate the velocity profile that the tumor cells may experience within the biomimetic platform. Toward this, we selected a horizontal plane 0.5 mm below the bottom of the transwell as the primary location for velocity analysis (Fig. 4(a)) to simulate the exchange between the tumor interstitium and the vessel. Our data show that the calculated velocity along this plane varied between 8.358x10^-7^ and 7.041x10^-4^ m/s with an average of 6.358x 10^-5^ m/s, while a maximum velocity of 0.004 m/s was achieved in the spouts of the LV-like biomimetic platform.

To simulate the constant oscillatory fluid shear forces that the lymphatic vessels in the body experience due to physical movement, the biomimetic platform is placed on an orbital shaker. The oscillatory forces applied by this orbital shaker induce a maximum wall shear stress of 1.83 dyne/cm^2^ for regular media and 2.31 dyne/cm^2^ for media supplemented with PEG, both within the range of shear stress observed in biological lymphatic fluid (0.64 - 12 dyne/cm^2^) [31].

Next, we generated a shear stress profile from the same stationary CFD model and observed that a maximum shear stress of 9.17x10^-4^ dyne/cm^2^ is reached along the surface of the transwell, while a lower maximum shear stress of 2.52x10^-4^ dyne/cm^2^ is experienced along the bottom of the biomimetic platform (Fig. 4(b)). Taken together, this platform named the 3D Tumor-Lymphatic Architecture Biomimetic (3D T-LAB) simulates the biomechanical properties of the tumor-lymphatic vessel interface and the lymphatic vessel.

### D. Lymphatic Endothelial Cells Promote Collective Invasion in the Tumor Lymphatic Interface

**Fig. 5.**
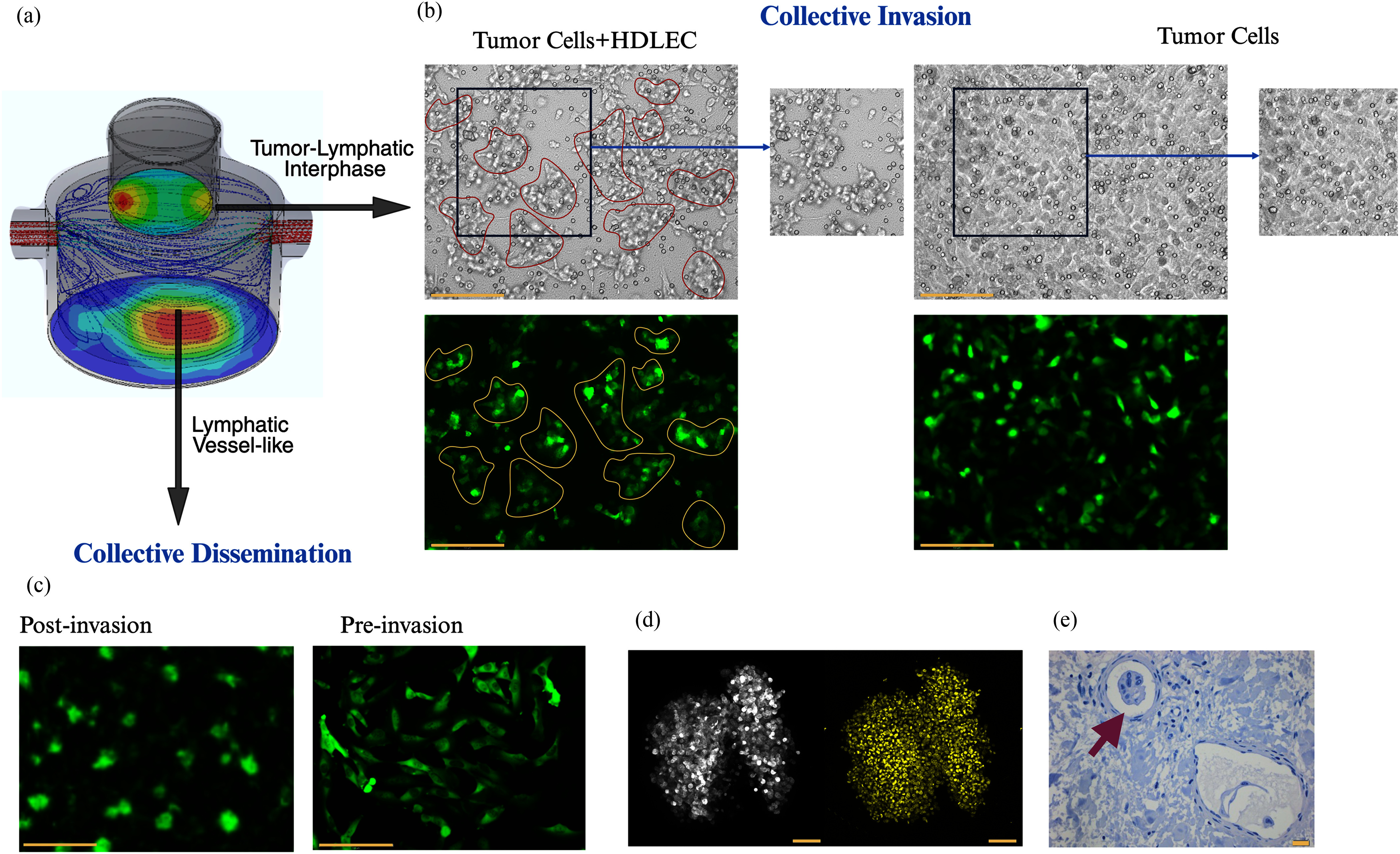
Biological application of the 3D T-LAB to access collective invasion and dissemination. (a) Fluid flow model of the T-LAB platform depicting the tumor-lymphatic interface and dermal lymphatic vessel. (b) Left panel: Co-culture of HDLEC cells were seeded first atop the transwell permeable membrane layered, followed by a tumor cell (SUM149-GFP-Luc) layer. Right panel: Tumor cells alone. Both conditions were assessed by imaging at 24-hour timepoint similar to Fig. 3. Bar represents 150 µm. The contouring shows collective phenotype in the co-culture conditions. (c) Collective Dissemination: To assess behavior and isolate invaded cells that are disseminating in the lymphatic vessel-like chamber. These can be expanded and compared with pre-invaded cells. Bar represents 150 µm. (d) Tumor emboli formation and live imaging using confocal microscopy where cells/GFP is shown in gray and nucleolus stained with Hoechst is shown in yellow. Representative image is maximum z-projection of 72 slices with step size of 1.27 µm. Bar represents 100 µm. (e) Arrow indicates tumor cell clusters/emboli in a lymphatic vessel of a deidentified IBC patient biospecimen.

To assess the tumor cell behavior traversing the tumor lymphatic interface, we compared tumor cells seeded in the 3D T-LAB [Fig. 5(a)] in the presence (Fig. 5(b)**-Left panel**) or absence (Fig. 5(b)**-Right panel**) of HDLEC co-culture conditions in the transwell. Live imaging of GFP-tagged IBC cells co-cultured with HDLEC show tumor cell clusters within 24 hours compared to the cells cultured alone (Fig. 5(b)**; Supplementary Fig. 1**). In both cases, cells invaded through the matrix within a 72-hour time point.

### E. Collective Dissemination of Tumor Cells in the Lymphatic Vessel-like Chamber

The tumor cells that have invaded in the lymphatic vessel-like bottom chamber show features of collective dissemination, with clustering morphology (Fig. 5(c)). Furthermore, confocal imaging of the invaded cells shows viable tumor cell clusters (Fig. 5(d)) similar to the diffusely migrating tumor emboli in the lymphatic vessels of patients with aggressive cancers like IBC (Fig. 5(e)). These findings indicate that HDLECs, in conjunction with the fluidic properties of the biomimetic platform create conditions that simulate tumor lymphatic interactions and the collective invasive phenotype.

## IV. Discussion

Collective metastasis enhances the ability of tumor cells to efficiently establish colonies in secondary locations, evade cell death, and evade detection by the immune system [32]. While circulating tumor cells are commonly seen in various cancers and are being investigated through genomic methods, isolating them in sufficient numbers for detailed morphological and phenotypic analysis can be a significant challenge. Herein, the 3D T-LAB developed aims to mimic the characteristics of dermal lymphatics and the interface between tumors and lymphatic vessels that allows live imaging and assessment of the behavior of cancer cells undergoing collective migration, invasion, and dissemination in a single platform. It achieves this by using a chamber design that supports a hydrogel mimicking the extracellular matrix (ECM), along with a monolayer of HDLEC on a basement membrane. Additionally, the system incorporates biomechanical factors such as pressure, lymphatic fluid flow, and shear stress, simulating the physiological conditions of the lymphatic environment. Mathematical and computational modeling along with cell-based experiments assessing these simulated features confirmed that the biomimetic model accurately represents a tumor lymphatic microenvironment. Furthermore, the conditions foster tumor cell clusters/emboli formation and collective invasion. Moreover, the 3D T-LAB’s straightforward and cost-effective manufacturing process makes it an economically viable model. Its versatility extends to various applications, including live imaging and the analysis of tumor cell collective migration and dissemination into the lymphatic vessel-like chamber, as well as changes in protein expression patterns. Comparing these data to changes observed in patients would attest to the 3D T-LAB’s relevance and provide valuable mechanistic insights.

The quantitative findings regarding pressure (increased by +1.38 mmHg), fluid flow (with a flow rate of 0.642 mL/min and velocity ranging between 8.358x10^-7^ and 7.041x10^-4^ m/s beneath the transwell), and shear stress (peaking at a maximum of 1.83 - 2.31 dyne/cm^2^) incorporated into the 3D T-LAB closely mirror the conditions observed in tumor environments. For instance, primary breast tumors exhibit increased interstitial pressure (rising to 15 mmHg ± 9 mmHg from a mean interstitial fluid pressure of 0 mmHg in normal breast tissue) [33]. Dermal lymphatic flow, which is pulsatile in nature, ranges from 5x10^-8^ mL/min to 2.3x10^-6^ mL/min, and oscillatory fluid shear stresses vary from 0.64 to 12 dyne/cm^2^. However, it is important to consider various aspects of the analysis methodology and acknowledge any limitations associated with these findings.

First, the analytical (5) used to determine the maximum shear stress due to the orbital shaker approximates the wall shear stress at the base of the biomimetic platform only. While it is reasonable for this to serve as an estimate of maximum shear stress present throughout the biomimetic platform, the shear stress experienced by cells in the transwell or during invasion into the bottom chamber remains unknown.

Second, the current CFD model is a stationary model and does not incorporate the motion of the orbital shaker. Since the shear stress produced by the orbital shaker was analytically calculated to be several orders of magnitude greater than the shear stress observed in the CFD fluid model, it is likely that the contribution of flow towards overall shear stress is greatly outweighed by the oscillatory motion and can be considered negligible. Similarly, the velocity of the fluid through the biomimetic platform may be affected by the orbital shaker. Current CFD models are limited in their ability to incorporate both the complex rotation of the orbital shaker and the necessary flow parameters, but further development of a model that more closely emulates the 3D T-LAB setup would be useful towards establishing a more detailed depiction of the biomimetic platform’s fluidic environment. Flow through the biomimetic chamber was also assumed as steady state despite the pulsatile nature of the peristaltic pump which mimics the pulsatile flow of the lymphatics.

Third, the established flow rate (0.642 mL/min) was faster than the rate needed to achieve similar fluid characteristics with the lymphatics. While dynamic similarity between the biomimetic platform and the lymphatic system cannot be fully achieved because the geometries are not identical, dimensional analysis to compute desired velocities and angular frequency, while accounting for the size discrepancy between the lymphatics and the 3D T-LAB system, was completed using dimensionless parameters like the Reynolds number and the Womersley number. Both geometries were approximated as cylinders and the biomimetic platforms’ calculated hydraulic diameter was applied. The used flow rate was ultimately limited by the speed settings of the selected peristaltic pump. Studies are ongoing to develop a customized peristaltic pump to achieve flow rates and velocities closer to the *in vivo* parameters and to optimize a multi-well 3D T-LAB with separate flow/well.

The biological insights gained from the 3D T-LAB system, using patient-derived cell lines, isogenic derivatives, and PDX models to mimic and study tumor emboli formation, collective migration, invasion, and dissemination mirrors clinicopathological features seen in patients and previous preclinical models [34], [35], [36], [37], [38]. This offers compelling evidence for the versatility of this system. Its potential applications are broad, including diagnostics, particularly for assessing the metastatic potential of tumor cells from core biopsies; drug screening to target metastatic cells; and generating new variants of metastatic cell lines for detailed investigations of tumor-microenvironment of any cancer type.

## V. Conclusion

Lymphatics have a critical role in the progression of cancer and patient prognosis, but as current understanding of this role is limited, it is necessary to create a pre-clinical model that enables further study of this route of invasion and dissemination dynamics. The lymphatic vessel-like biomimetic platform presented here is an innovative model of a dermal lymphatic vessel and surrounding tumor microenvironment through its simulation of fluid flow, increased pressure, and shear stress corresponding to physiological parameters. Furthermore, experiments utilizing this biomimetic platform have shown that the simulated biomechanical parameters support hallmarks of inflammatory breast cancer like time-dependent invasion of tumor cells and the formation of tumor emboli in the presence of HDLECs. Ongoing studies include optimizing conditions in the 3D T-LAB to isolate the cells that have invaded into the lymphatic vessel like chamber and clonally expand for molecular characterization and development of new metastatic cell lines. In addition, the 3D T-LAB platform has the potential to be applied in broad, far-reaching investigations including but not limited to tumor-lymphatic interactions, lympho-vascular invasion, and drug-screening. Ultimately, this places the 3D T-LAB as a critical tool in predicting metastatic behavior at diagnosis, identifying biomarkers, and for testing treatment strategies.

## Supplementary Materials

Supplementary Table 1, Supplementary Table 2, Supplementary Methods.

Supplementary Materials
