# Supplementary material for: 3D Engineered Biomimetic Platform for Characterization of Collective Invasion, Tumor Emboli Formation, and Lymphatic Dissemination
Source: IEEE Open J Eng Med Biol. 2026 Feb 23;7:119–27. doi: 10.1109/OJEMB.2026.3666977 (PMC13068115; doi:10.1109/OJEMB.2026.3666977)
Supplement: Supplementary Materials [file supp1-3666977.docx]

**Supplementary Table 1.** The properties of the media used for cell culture in the 3D T-LAB measured at 37°C compared with reported properties of lymphatic fluid *in vivo.* *Calculated using the dynamic viscosity and density reported by Moore and Bertram (2018)

|  | **Bioreactor** | | ***In Vivo*** |
| --- | --- | --- | --- |
|  | PEG Media | Regular Media | Lymphatic Fluid |
| Kinematic Viscosity (cSt) | 1.1356 | 0.7196 | 1.22* |
| Dynamic Viscosity (cP) | 1.1308 | 0.7124 | 1.23  (Moore and Bertram, 2018) |
| Density (g/cm^3^) | 0.9957 | 0.9899 | 1.0097  (Moore and Bertram, 2018) |

**Supplementary Table 2.** Unit equivalency chart.

| **Unit** | **Equivalent Unit** | |
| --- | --- | --- |
| 1 cP | 0.01 g/cm-s | 0.001 Pa*s |
| 1 dyne/cm^2^ | 0.1 Pa |  |
| 1 cSt | 1 mm^2^/s |  |
| 1 g/mL | 1 g/cm^3^ |  |
| 1 rpm | $\frac{\pi}{30}$ rad/s | ≈ 0.1047 rad/s |

Supplementary Methods

## Cell Culture

Human breast carcinoma cells MDA-MB-231 (ATCC), human inflammatory breast cancer (IBC) patient-derived treatment naïve cell line SUM149 (triple negative), and patient derived explant (PDX) cell line MDA-IBC3 (negative for hormone receptors but overexpress human epidermal growth factor receptor 2/HER2; kindly gifted by Dr. Woodward) [22]. Adult human dermal lymphatic endothelial cells (HDLEC) (Promocell, Germany) were cultured in the Endothelial Cell Growth Medium MV 2 (catalog #C-12217;) with supplement mix (catalog #C-22022) according to manufacturer instructions. All cell cultures utilized in this study were maintained at 5% CO_2_ atmosphere and 37°C. All cell lines underwent recent STR profiling, and low passage numbers were used for all experiments.

Imaging

After 24, 48, and 72 hours, z-stack images of the transwell membrane were taken using the automated mode of the 20X objective in an EVOS M7000 inverted microscope (ThermoFisher, Cincinnati, OH) to track the seeded tumor cells invading through the collagen layer in the membrane.

For emboli imaging experiment, the collected tumor emboli were centrifuged at 1600 rpm for 4 minutes, resuspended in 100 μL of cold DPBS, and stained with Trihydrochloride, Trihydrate/Hoechst 33342 (ThermoFisher, Bend, OR) for 30 minutes and imaged using a Zeiss 880 inverted confocal microscope (Zeiss, Germany) with a Märzhäuser linearly encoded x,y stage, and a 20X (NA 0.8) with 0.6 optical zoom apochromatic objective (Montero Llopis, Senft et al. 2021).

Quantitative Viability Assays

For quantitative viability experiments, media atop the collagen layer were collected at different time points in a microcentrifuge tube without touching the membrane in the upper chamber. The insert was rinsed gently with 0.1 mL PBS, and the rinse liquid was transferred the same tube as the collected media. Then, 0.1 mL trypsin/EDTA was added, and inserts were incubated at 37°C for 10-15 minutes to break up and dissolve the collagen layer and any cells attached to the transwell. Dissociated cells were then transferred to the same tube as the media and rinse liquid. Collected cell solutions were centrifuged at 1600 rpm for 4 minutes, and the pellet was resuspended in 100 μL of cold DPBS. A 10 µL aliquot of the cell mixture was resuspended in an equal volume of 0.4% trypan blue solution and 10 μL of the resultant mixture was subjected to cell count (blue vs. unstained cells) using a hemocytometer. Percent cell viability was assessed as # non-stained / total cells (x100).
